# Distribution of the Long-Horned Beetle, *Dectes texanus,* in Soybeans of Missouri, Western Tennessee, Mississippi, and Arkansas

**DOI:** 10.1673/031.010.14138

**Published:** 2010-10-11

**Authors:** Kelly V. Tindall, Scott Stewart, Fred Musser, Gus Lorenz, Wayne Bailey, Jeff House, Robert Henry, Don Hastings, Milus Wallace, Kent Fothergill

**Affiliations:** ^1^ University of Missouri Division of Plant Sciences, Delta Research Center P.O. Box 160, Portageville, MO 63873; ^2^ The University of Tennessee, 216 West Tennessee Research and Education Center, 605 Airways Boulevard, Jackson, TN 38301; ^3^ Mississippi State University Dept. of Entomology and Plant Pathology, P.O. Box 9775, Mississippi State, MS 39762; ^4^ University of Arkansas, 2001 Highway 70 E. Box 357, Lonoke, AR 72086; ^5^ University of Missouri Extension, 1-87 Agriculture Building, University of Missouri-Columbia, Columbia, MO 65211; ^6^ University of Missouri New Madrid County Extension Center, 420 Mott Street, New Madrid, MO 63869; ^7^ Robert Henry Seed Company, P.O. Box 279, New Madrid, MO 63869; ^8^ Soybeanscouting.com, 3299 West 520^th^ Road, East Prairie, MO 63845; ^9^ Conservation Seeding and Restoration, Inc., 506 Center Street West, Kimberly, ID 83341

**Keywords:** *Glycine max*, distribution

## Abstract

The long-horned beetle, *Dectes texanus* LeConte (Coleoptera: Cerambycidae), is a stem-boring pest of soybeans, *Glycine max* (L.) Merrill (Fabales: Fabaceae). Soybean stems and stubble were collected from 131 counties in Arkansas, Mississippi, Missouri, and Tennessee and dissected to determine *D. texanus* infestation rates. All states sampled had *D. texanus* present in soybeans. Data from Tennessee and Arkansas showed sample infestations of *D. texanus* averaging nearly 40%. Samples from Missouri revealed higher infestation in the twelve southeastern counties compared to the rest of the state. Data from Mississippi suggested that *D. texanus* is not as problematic there as in Arkansas, Missouri, and Tennessee. Infestation rates from individual fields varied greatly (0–100%) within states. In Tennessee, second crop soybeans (i.e. soybeans planted following winter wheat) had lower infestations than full season soybeans. A map of pest distribution is presented that documents the extent of the problem, provides a baseline from which changes can be measured, contributes data for emergency registration of pesticides for specific geographic regions, and provides useful information for extension personnel, crop scouts, and growers.

## Introduction

The long-horned beetle, *Dectes texanus* LeConte (Coleoptera: Cerambycidae), was first reported as a pest of soybeans *Glycine max* (L.) Merrill (Fabales: Fabaceae) in 1968 in Beaufort County, North Carolina (Falter 1969) and in New Madrid and Dunklin Counties, Missouri (Hatchett et al. 1973). Falter (1969) also noted *D. texanus* to be present in Arkansas soybeans. Since that time it has been reported as a soybean pest in Illinois, Kansas, Kentucky, Nebraska, Tennessee, and Texas. *Dectes texanus* is also a pest of the sunflower (*Helianthus annuus* L.) (Rogers 1977; Bushman and Sloderbeck 2007; Michaud and Grant 2009).

*Dectes texanus* has one generation per year (Falter 1969; Hatchett et al. 1975), and the partially grown larvae overwinter inside the stem of the host plant (Hatchett et al. 1975). Adults are typically sampled with sweep nets in soybeans, however, there is little information on the adequacy of this method, and there are no models that relate larval infestation to adult numbers. The methods described in this paper are adequate for detecting infestation at agriculturally relevant levels.

Females oviposit in petioles and damage from larval feeding causes the petioles to turn yellow, wilt, and eventually drop (Hatchett et al. 1975). When petioles drop an entrance hole is evident where the larva entered the main stem of the soybean plant. However, yellow petioles and main stem larval entrance holes are not often noticed, therefore, infestations are more reliably detected by splitting stems. When *D. texanus* damage is noticed in soybeans, it may take the form of a wilted top third of the plant and is often misdiagnosed as another ailment, especially sudden death syndrome caused by *Fusarium solani* (Mart.) Sacc. f. sp.*glycines* (J. House, R. Henry, personal observation). Financial losses attributed to this insect in soybeans are due primarily to lodging (Hatchett et al. 1975; Campbell and VanDuyn 1977), but physiological yield losses may also occur (Richardson 1975; Davis et al. 2008; KVT, unpublished data).

Lingafelter (2007) states that plants in the genera *Ambrosia, Gaillardia, Helianthus, Solidago,* and many other herbaceous plants are suitable hosts for *D. texanus.* These include cocklebur, *Xanthium strumarium,* crested anoda, *Anoda stata,* cowpea, *Vigna unguiculata* and common and giant ragweeds, *A. artemisiifolia* and *A. trifida* (Piper 1978). *Dectes texanus* is also widely distributed (Rice and Enns 1981; MacRae 1993; Yanega 1996; Lingafelter 2007), likely due to the wide distribution of its host plants in North America. Mapping the range of *D. texanus* in soybeans and the degree of soybean infestation at county level provides important data on geographical occurrence and serves as a baseline from which future range expansion in soybeans can be assessed.

## Material and Methods

**Figure 1.**
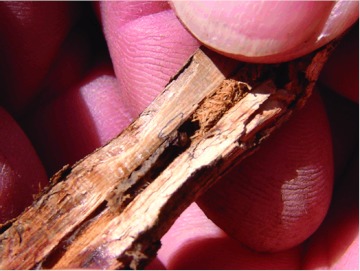
*D. texanus* tunneled soybean stem, frass plug present with a dead larva. High quality figures are available online.

Researchers in multiple states collected data to determine the distribution and infestation rate of soybean by *D. texanus* in counties in Arkansas, Mississippi, Missouri, and Tennessee. Soybean stems were dissected (whole soybean plants and post-harvest stubble) to determine the infestation rates of individual fields (Hatchett et al. 1975). Soybeans were considered infested if a *D. texanus* larva and/or a frass plug was found within the stem or stubble (Figure 1). Approximately 30–50 stems were sampled per field. Whereas 30–50 stems may not accurately represent a 16 ha field with a plant population of 250,000 plants/ha, the authors feel that in a field with a moderate to high rate of infestation, *D. texanus* can be detected using this methodology. All samples were processed in a similar manner. Descriptive statistics were generated for all counties sampled within a state using PROC MEANS in SAS (SAS 1998).

### Missouri

Sixty-nine fields planted to soybeans in 2007 were sampled in seven counties (Table 1) in southeast Missouri to determine presence of *D. texanus.* Fields were sampled opportunistically (i.e. as encountered within sampling forays) with care taken to avoid geographic clustering within counties. Approximately 50 soybean stems were collected for dissection to determine the presence of *D. texanus* in each field. Fields from 2007 were sampled late March through April 2008.

In 2008, 401 fields planted to soybeans in all 85 counties with more than 600 ha of soybean production (NASS 2009) were sampled post-harvest, as in the previous year, with the exception that sampling occurred from December 2008 through March 2009. Two additional counties (with < 600 ha of soybean production) were sampled as soybean fields were encountered within these counties during sampling forays (Table 2).

A random subset of larvae from the 2007 collections (n = 479) were diet (singular) (Hatchett et al. 1973) in an insect rearing room (16:8, 24° C). The artificial diet of Hatchett et al. (1973) was modified to reflect currently available diet ingredients (Product #F9703B, Bio-Serv, www.bio-serv.com). Adults were identified (Linsley and Chemsak 1995) to confirm larval identification (TC MacRae).

### Tennessee

Forty-five fields planted to soybeans in 2007 were sampled as previously described, except that 30 plants per field were sampled instead of 50 in each of nine counties (Table 3). Fields were sampled late August through September 2007. Data were collected for infestation and whether the soybeans were first or second crop (i.e. beans following winter wheat). County means are reported in tables.

**Table 1.**
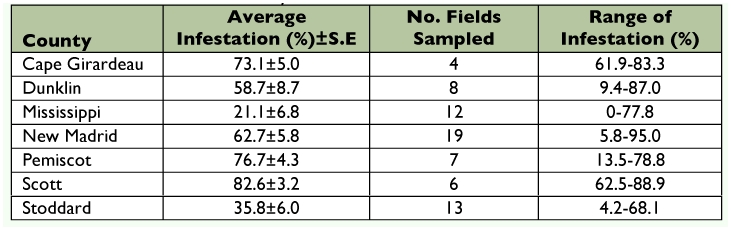
Percent infestation of *Dectes texanus* in soybeans, southeast Missouri, 2007.

**Table 2.**
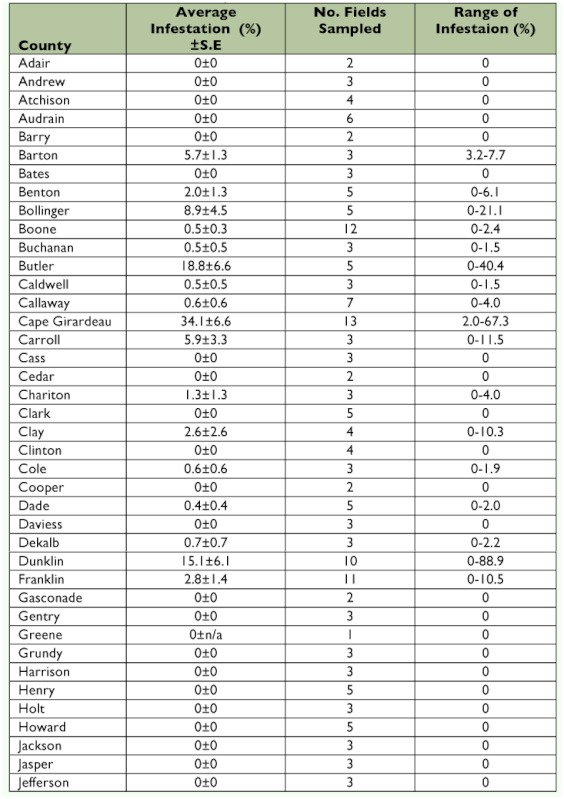
Percent infestation of *Dectes texanus* in soybeans, Missouri, 2008.

**Table:**
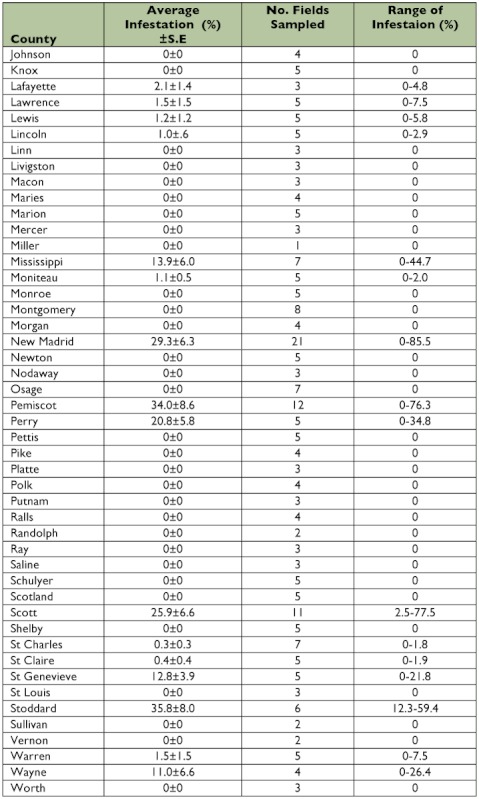

**Table 3.**
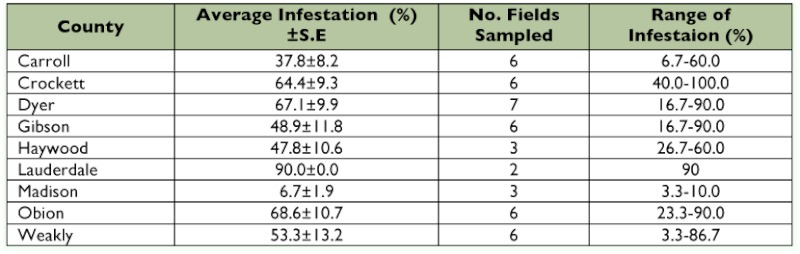
Percent infestation of *Dectes texanus* in soybeans from select counties in Tennessee, 2007.

**Table 4.**
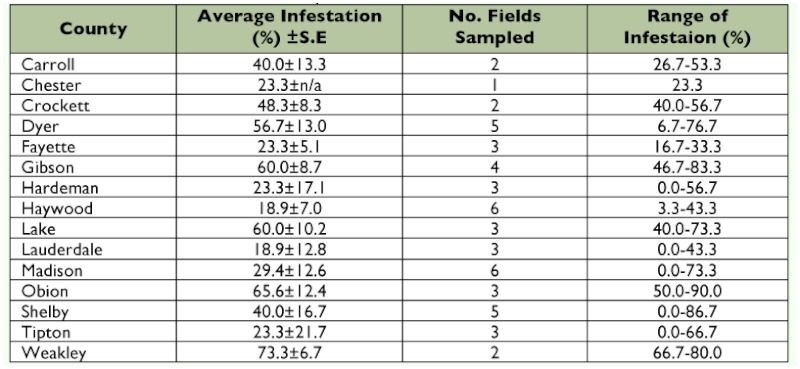
Percent infestation of *Dectes texanus* in soybeans from select counties in Tennessee, 2008.

**Table 5.**
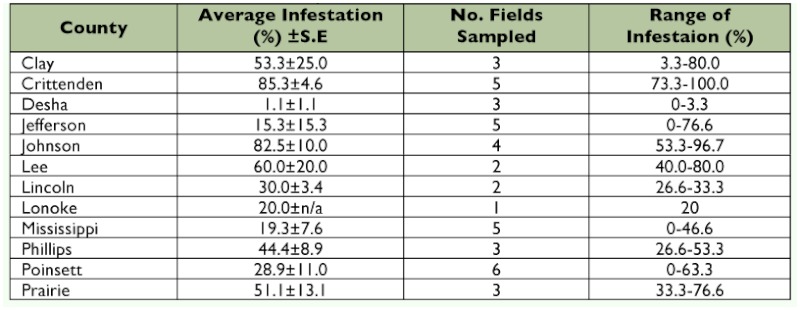
Percent infestation of *Dectes texanus* in soybeans from select counties in Arkansas, 2008.

**Table 6.**
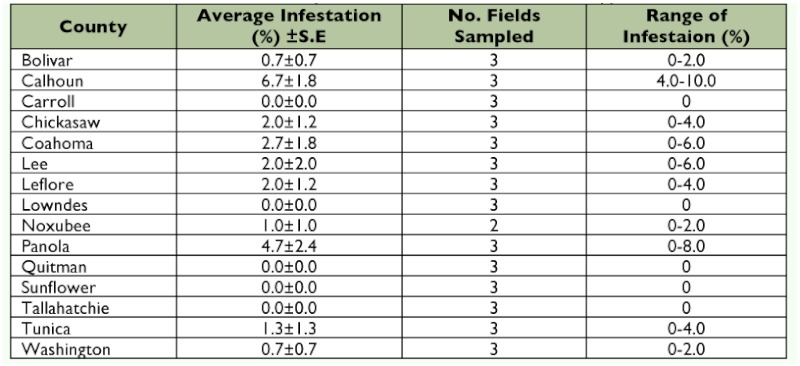
Percent infestation of *Dectes texanus* in soybeans from select counties in Mississippi, 2008.

In 2008, 51 fields planted to soybeans were sampled in fifteen counties (Table 4) in October 2008. Data were collected for infestation and whether soybeans were the first or second crop, as in 2007. Data for both 2007 and 2008 full season and second crop soybean plantings were analyzed by ANOVA using PROC MIXED in SAS (SAS 1998) to determine if the infestation rates between the two plantings differed. The variable ‘planting date’ (i.e. first or second crop beans) was treated as a fixed effect and ‘year’ was used in the RANDOM statement.

**Figure 2.**
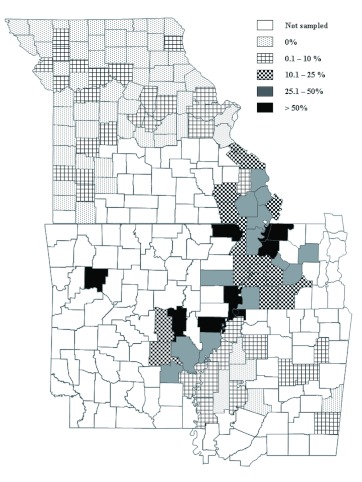
Distribution of *Dectes texanus* in Arkansas, Mississippi, Missouri, and Tennessee in 2008 with rates of infestation by county. High quality figures are available online.

### Arkansas

Forty-five fields planted to soybeans in 2008 were sampled as described above, 30 plants per field. The fields were located in thirteen counties (Table 5) and were sampled late from August through early October 2008.

### Mississippi

Forty-seven fields planted to soybeans in 2008 were sampled, 50 plants per field. The fields were located in sixteen counties (Table 6) and were sampled during March of 2009.

## Results

All states sampled had *D. texanus* present in soybeans. Infestation of soybean samples by *D. texanus* was similar between southeast Missouri and northwest Tennessee in 2007. Based on our samples in 2008, Arkansas and Tennessee had severe infestations of *D. texanus* in soybeans.

### Missouri

A total of 3242 stems were examined in 2007 and an average of 51.0 ± 3.4% of these stems were tunneled by *D. texanus.* Infestations of individual field samples ranged from 0–95% (Table 1). All larvae reared to adulthood were identified as *Dectes texanus* (n = 479).

A total of 21,814 stems were sampled in 2008. The statewide average infestation of these stems was 6.7 ± 0.8% and infestations of fields ranged from 0–85% (Table 2, Figure 2). There was a trend in twelve counties in southeast Missouri (Ste. Genevieve, Perry, Cape Girardeau, Bollinger, Wayne, Butler, Scott, Stoddard, Mississippi, New Madrid, Pemiscot, and Dunklin counties) of higher infestations than the rest of the state. Carroll and Barton counties also had notable infestation and are located outside the southeastern region.

### Tennessee

A total of 1350 stems were sampled in 2007 and the statewide average infestation was 54.5 ± 3.4%. Every field sampled had *D. texanus* present, but infestations of individual field samples ranged from 3–100% (Table 3).

A total of 1530 stems were sampled in 2008 and the statewide average infestation was 39.3 ± 4.0%. Infestations of fields ranged from 0– 90% (Table 4, Figure 2).

*D. texanus* infestation rates in second crop beans were compared to full season soybeans with data from both seasons pooled. The infestation rate of *D. texanus* in full season soybeans (51.9 ± 3.4%) was significantly greater than in second crop soybeans (39.0 ± 5.2%; F = 12.57; df = 1, 93; P = 0.0006).

### Arkansas

A total of 1350 stems were sampled in 2008 and the statewide average infestation was 41.4 ± 5.0%. Infestations of individual field samples ranged from 0–100% (Table 5, Figure 2).

### Mississippi

A total of 2350 stems were sampled in 2008 and the statewide average infestation was 1.5 ± 0.4%. Infestations of individual field samples ranged from 0–10% (Table 6, Figure 2).

## Discussion

The 2008 samples reveal a ‘hot spot’ of *D. texanus* infestation where Tennessee, Missouri, and Arkansas border one another (Figure 2). Infestation rates from Mississippi and the northern area of Missouri suggest that *D. texanus* is not as problematic in those areas. However, the degree of infestation varied greatly among samples within each state.

Field to field variations in infestation rate were observed within each state. Fields known to be second crop soybeans had lower infestations than full season soybeans in Tennessee. This trend has also been noted for second crop beans in Arkansas and Missouri (GL, KVT, personal observation). There are also reports that some varieties are resistant to *D. texanus* injury (Richardson 1975, GL unpublished data), however, there have not been any field studies to determine if varietal preference can affect infestation rate over a large scale. Multiple pyrethroid applications can reduce infestation by *D. texanus* (Sloderbeck et al. 2004) and this may partially explain why Mississippi has lower rates of infestation. Mississippi has additional insect pressures, such as soybean looper [*Pseudoplusia includens* (Walker)] and bean leaf beetle [*Ceratoma trifurcata* (Forster)], in soybean that trigger more frequent insecticide applications than the other areas we sampled (Musser et al. 2009). Burying soybean stubble 5 cm or more negatively affects larval survival and adult emergence (Campbell and Van Duyn 1977) and Mississippi has a relatively high percentage of fields that are tilled. Tillage buries soybean stubble which reduces overwintering survival of *D. texanus* and may contribute to low infestations in Mississippi. Several individual fields in each state had low infestations, but it is not clear if this was due to second crop planting, resistant varieties, management practices, field-specific environmental parameters, or random effects. Additionally, it seems likely that low level *D. texanus* infestations could have escaped detection in this study given the small numbers of stems sampled in each field. Further research is needed to elucidate why some fields have lower infestations than others.

Twenty counties in Missouri and five counties in Mississippi were found to have *D. texanus* populations not previously documented in any host plant (Rice and Enns 1981; MacRae 1993) (Terence Schiefer, personal communication). Additionally, specimens of *D. texanus* in the Mississippi Entomological Museum have collection data stating an association with soybeans in two counties where the pest was not detected in this study (Sunflower and Leflore) and three counties not sampled (Pontotoc, Issaquena, and Pearl River) (Terence Schiefer, personal communication). This indicates that use of soybean as a larval host for *D. texanus* is widespread, and may be increasing. Bushman and Sloderbeck (personal communication) also found an increasing geographic range of soybean feeding by *D. texanus* in Kansas. The mapping of pest occurrence within crops documents geographic range, provides a baseline against which changes can be detected, contributes data for emergency registration of pesticides for specific geographic regions, and provides useful information for extension personnel, crop scouts, and growers.

While little is known about the dispersal of *D. texanus,* other members of the subfamily Lamiinae are known to have a relatively small range of dispersal. *Acalolepta vastator* (Newman) disperses 105 m per year (Goodwin et al. 1994); *Anoplophora glabripennis* (Motschulsky) is capable of dispersing up to more than 1400 m (Smith et al. 2001). The dispersal of *Monochamus alternatus* Hope was less than 100 m but dispersal was affected by several factors including the density of available hosts and the size of the population of *M. alternatus* emerging (Togashi 1990). A distribution study of *D. texanus* was conducted in Kansas in the late 1990s and repeated in 2008. Results from the survey in 2008 revealed that there was a significant increase in infestation rates in areas where *D. texanus* were detected at low levels in the late 1990s (Buschman and Sloderbeck 2010). This suggests that over time the rate of infestation of soybean by *D. texanus* may increase in areas with low infestation rates.

Currently, there is no effective method of sampling *D. texanus* adults or correlating adult numbers to larval infestation. It is advisable to sample soybean fields near harvest to be aware of the potential for lodging based upon rate of infestation and ensure soybeans are harvested in a timely manner. Growers not able to sample prior to harvest have the option of using the methods described in this manuscript to determine infestation levels post-harvest. In areas of high infestation, a grower may consider planting an alternate crop. However crop rotation, unless conducted on a large scale, is not likely to be effective because *D. texanus* is able to migrate from neighboring fields.

It was postulated that *D. texanus* convergently evolved the ability to utilize soybean in multiple locations, which would explain the many states in which soybean feeding has been observed (Michaud and Grant 2005). Given the wide plant taxonomic (Piper 1978) and geographic ranges within which *D. texanus* lives and feeds, utilization of soybeans as a larval host plant may have been relatively easy for this insect. More research is needed to fully understand distribution, biology, and impact of *D. texanus* populations in soybeans.
